# Surface EMG Statistical and Performance Analysis of Targeted-Muscle-Reinnervated (TMR) Transhumeral Prosthesis Users in Home and Laboratory Settings

**DOI:** 10.3390/s22249849

**Published:** 2022-12-14

**Authors:** Bingbin Wang, Levi Hargrove, Xinqi Bao, Ernest N. Kamavuako

**Affiliations:** 1 Department of Engineering, King’s College London, London WC2R 2LS, UK; 2 Center for Bionic Medicine, the Shirley Ryan Ability, Chicago, IL 60611, USA; 3 Department of Physical Medicine and Rehabilitation, Feinberg School of Medicine, Northwestern University, Chicago, IL 60611, USA; 4 Faculté de Médecine, Université de Kindu, Site de Lwama II, Kindu, Maniema, Congo

**Keywords:** pattern recognition, electromyography, myoelectric control, TMR, separability index

## Abstract

A pattern-recognition (PR)-based myoelectric control system is the trend of future prostheses development. Compared with conventional prosthetic control systems, PR-based control systems provide high dexterity, with many studies achieving >95% accuracy in the last two decades. However, most research studies have been conducted in the laboratory. There is limited research investigating how EMG signals are acquired when users operate PR-based systems in their home and community environments. This study compares the statistical properties of surface electromyography (sEMG) signals used to calibrate prostheses and quantifies the quality of calibration sEMG data through separability indices, repeatability indices, and correlation coefficients in home and laboratory settings. The results demonstrate no significant differences in classification performance between home and laboratory environments in within-calibration classification error (home: 6.33 ± 2.13%, laboratory: 7.57 ± 3.44%). However, between-calibration classification errors (home: 40.61 ± 9.19%, laboratory: 44.98 ± 12.15%) were statistically different. Furthermore, the difference in all statistical properties of sEMG signals is significant (*p* < 0.05). Separability indices reveal that motion classes are more diverse in the home setting. In summary, differences in sEMG signals generated between home and laboratory only affect between-calibration performance.

## 1. Introduction

Limb amputation refers to the remove all or part of an upper or lower extremity. When people lose their upper limbs, many activities of daily living are significantly limited, as they interact with their surroundings and perform sophisticated tasks with their hands. According to hand and upper-limb reconstruction statistics provided by the NHS [1], the total number of amputations in the United Kingdom is estimated to be 250,000, with 10,000 increments per year. One out of four people with limb loss is an upper-limb amputee.

Prostheses aim to replace lost limbs and restore functionality. Myoelectrically controlled prostheses are state-of-the-art devices that intuitively interpret muscle signals to control the prostheses. In developing control schemes for myoelectrically controlled prostheses, control schemes have evolved from the initial on–off control to the two most popular methods, namely proportional amplitude control and pattern-recognition-based control [2]. Conventional proportional control schemes with two electrodes control the prosthesis with one degree of freedom and vary the control voltage according to the amplitude of the sEMG signals, providing robust performance but limited functionality [3]. Both control schemes can provide reasonable controllability for prostheses. Despite advancements in myoelectric control of prostheses, the prosthetic abandonment rate has not changed significantly since 2007 [4]. It has been estimated that about 52.18% of amputees abandon their prostheses due to concerns about comfort and functionality.

Pattern-recognition-based control schemes increase functionality by mapping decoded sEMG signals to different motion patterns, but their ability to predict movements accurately deteriorates over time [5,6,7,8]. This is mainly caused by the limited amount of data used to train the systems, which does not include variability in sEMG caused by intrinsic and extrinsic factors such as muscle fatigue [9], skin impedance [10], electrode shift [11], mutual adaptation [12], etc. Extensive training data sets that include example data representative of these conditions are challenging to collect clinically. Consequently, tools such as mobile applications and device-supervised training routines have been developed to allow users to recalibrate their PR control systems. Whereas recalibration can be performed quickly, the necessity to do so should be reduced as much as possible and used as a tool to personalize control rather than accommodate factors such as poor socket fit. The need to excessively recalibrate the device could lead to an increased possibility of abandonment of the prosthesis.

Recently, pattern-recognition-based control has become a viable option for clinical application due to the great promise of improved dexterity and performance [13]. Recent studies have focused on improving the robustness of prosthetic control systems through the simulation of potential clinical influence factors to transfer laboratory results to the clinic. Samuel et al. [14] suggested using the dual-stage sequential method, hybrid strategy, and multiscenario strategy based on accelerometer mechanomyography to mitigate the effect of mobility. The results show a significant reduction in classification error compared with other traditional classifiers. To improve pattern recognition performance under force variation, Islam et al. [15] proposed an improved time-domain feature extraction method, achieving 97.93% accuracy for seven hand gestures of nine amputees. Gigli et al. [16] used a dynamic training protocol to reduce errors caused by different limb positions. These trained systems outperformed statically trained systems by a significant margin. In addition to using additional data, extracting invariant features, or training the system with a novel protocol, postprocessing can also improve system reliability. To enhance the performance of clinical classification, Bao et al. [17] developed convolutional neural networks (CNNs) with confidence scores for rejecting low-confidence classification results. In online testing, their proposed method achieved an average error of 9.75% lower than a CNN based on majority voting and an original CNN.

Although the above studies achieved promising results, these results remain in the context of the laboratory rather than a clinical or home setting. Home trials have only been conducted in a handful of research studies. Osborn et al. [18] conducted a nine-week home trial case study with an amputee with extensive experience using prosthetics to understand how pattern-recognition-based control prosthetic systems are incorporated into daily life. Furthermore, Simon et al. [19] compared user performance with pattern recognition control and direct control in eight-week home trials. Their study and the study conducted by Resnik et al. [20] provided insight into the use of different motion patterns in a home compared to a laboratory. However, these studies did not provide a deep analysis of the divergence of the sEMG in different contexts. There is a lack of knowledge about the usability of sEMG in the home versus laboratory settings. The open question is how prosthesis users perform in a laboratory relative to home. In these two contexts, the quality of control of prostheses might relate to the amputees’ motivation or awareness of the use of prostheses. There could be a potential difference in EMG signals and corresponding signal qualities. Hence, this study aims to compare the statistical properties of sEMG signals and quantify the difference between the calibration data (6–8 weeks) collected in home and laboratory setting using separation indices, repeatability indices, and correlation coefficients. Subsequently, we evaluate whether these metrics can be used to predict the usability of calibration data. In this study, we provide deep insight into the statistical properties sEMG signals and analyse the feature space distribution to evaluate the calibration quality of sEMG signals in home and laboratory contexts.

## 2. Materials and Methods

### 2.1. Data Source

Surface EMG signals were obtained from [21], acquired from eight targeted-muscle-reinnervated (TMR) transhumeral amputees with myoelectric prostheses using experience over six to eight weeks at home and in the laboratory. However, only data for seven participants were available to us; one contained home trial data, and another had a failure channel. Hence, we used the data of five participants in this study. The participants used custom-fabricated prostheses; a Boston Digital Elbow (Liberating Technologies Inc., Holliston, Massachusetts, USA), a Motion Control Wrist Rotator (Motion Control Inc., Salt lake City, Utah, USA), and a single-degree-of-freedom terminal device (a powered split hook or hand). The prostheses were embedded with eight stainless-steel electrodes sampling at 1000 Hz. These eight electrodes were grid-arranged [22] and placed on the wall of the prosthesis liner.

Before and after the home trial, several tests were performed in the laboratory to evaluate the prosthetic control performance of each participant. The goal was to identify optimal electrode sites inside the socket and make the amputee confident about using the device. The user was then included in the trial and sent home with the device. During the home trial, participants were instructed to control the prosthesis to perform activities of daily living and to record the use frequency and activities performed using the prosthesis. Calibration sessions at home were at the discretion of the participants. They could calibrate after donning or any time they noticed a decrease in performance. On the other hand, laboratory calibration sessions were conducted as instructed by the occupational therapist during laboratory visits throughout the trial.

In each calibration, seven movements were recorded, including elbow flexion, elbow extension, wrist pronation, wrist supernation, hand open, chunk grip, and rest. Except for rest, each calibration motion was supposed to be performed twice, lasting three seconds each. After each calibration, sEMG signal data were stored in the memory of the embedded controller so that prosthesis usage data could be accessed after the home or laboratory trial. We used the calibration data of the whole 6–8 weeks of home and laboratory trials. Table 1 shows calibration times for each participant. In addition, because the number of calibrations varies in the laboratory and home, we chose equal calibration times for the laboratory and home setting based on the side with fewer calibrations. We balanced the time of laboratory calibrations before and after the home trial. The selected data were as close in time as possible to minimize the effect of time, which could cause different body conditions, as well as familiarity with control of the prosthesis, resulting in different EMG signals.

### 2.2. Statistical Properties Calculation

We decided to describe raw sEMG signals using the following statistical properties to understand how the signals differ from home to the laboratory. Then, we averaged all calculated statistical properties of overall channels and motions for each calibration of each participant.

1. Root Mean Square (RMS)

(1) $$RMS=\sqrt{\frac{1}{n}\underset{i}{\sum }x_{i}^{2}}$$

where n is the number of samples, and $x_{i}$ is the amplitude of sample i.

2. Mean Frequency (MeanF) [23]

(2) $$MeanF=\frac{\sum_{i=0}^{M}P_{i}*f_{i}}{\sum_{i=0}^{M}P_{i}}$$

where M is the number of frequency bins, $f_{i}$ is the frequency of the spectrum at bin i, and $P_{i}$ is the power spectrum at bin i.

3. Median Frequency (MedF) [23]

(3) $$\sum_{j=1}^{MedF}P_{i}=\sum_{j=MedF}^{M}P_{i}=\frac{1}{2}\sum_{i=1}^{M}P_{i}$$

where $P_{i}$ is the power spectrum at bin i, and M is the number of frequency bins. The total power spectra are divided into two equal parts at the median frequency.

4. Variance

(4) $$Variance=\frac{1}{n-1}\sum {\left(x_{i}-\bar{x}\right)}^{2}$$

where $x_{i}$ is the amplitude of the signal at sample point i, $\bar{x}$ is the mean amplitude of sEMG signals, and n is the number of samples.

### 2.3. Signal Processing and Feature Extraction

The obtained sEMG signals were processed using MATLAB R2020b. We filtered the EMG signals between 20 and 500 Hz using a fourth-order Butterworth filter. Subsequently, filtered signals were segmented using overlapping windows of 200 ms, each with 30 ms increments. Hudgin’s feature set [24] with Willison amplitude was extracted in each window.

### 2.4. Calibration Quality Quantification

In our previous research [25], we demonstrated that quantification of feature change could effectively reflect how sEMGs change under time effect. Hence, quantifying the feature space variation could be critical to evaluating changes in calibration data. We tested four separability indices, one repeatability index, and two correlation coefficients as signal quality quantification metrics.

#### 2.4.1. Separability Indices

Separability indices between each motion were used to measure the diversity of each motion pattern in feature space based on statistical criteria for each calibration. These separability indices were related to the combination of within- and between-class information to describe the classifiability of calibration data. Because some methods are used to evaluate the separability between two classes, we calculated these indices between each motion class (i.e., there were K = x two-class combinations) and averaged them for single calibration data. In this study, we used the following four separability indices:

- Davies-Bouldin index (DBI) [26]

The DBI measures the worst-case separability of neighbouring classes in feature space by averaging the highest magnitude of overlap among them. Hence, a lower value of DBI indicates higher class separability. Equations (5)–(7) illustrate how it is computed:

(5) $$S_{h}=\sqrt{\frac{1}{N_{h}}\overset{N_{h}}{\underset{i=1}{\sum }}{\left(x_{i}-\mu_{h}\right)}^{T}\left(x_{i}-\mu_{h}\right),\;}x_{i}\in C_{h}$$

(6) $$D_{hl}=\sqrt{{\left(\mu_{h}-\mu_{l}\right)}^{T}\left(\mu_{h}-\mu_{l}\right)}\;$$

(7) $$R_{hl}=\frac{S_{h}+S_{l}}{D_{hl}}\;DBI=\frac{1}{K}\overset{K}{\underset{k=1}{\sum }}max{\;}_{h\neq l}\left(R_{hl}\right)$$

where $S_{h}$ is the diversity of features within a class, $C_{h}$ is the $h^{th}$ class, $C_{l}$ is the $l^{th}$ class ($C_{h}\neq C_{l}$), $N_{h}$ is the number of feature vectors in the $h^{th}$ class, $x_{i}$ is the $i_{th}$ feature vector in the $h^{th}$ class, *$D_{hl}$* is the similarity between classes, $\mu_{h}$ is the mean of the feature vector in the $h^{th}$ class, $R_{hl}$ combines $D_{hl}$ and $S_{h}$ to measure the overlap between two classes, and K is the number of pairs of classes.

- Simplified Silhouette value (SS) [27]

SS is a computationally efficient version of the silhouette value. It analyses the consistency of each point in its class and the diversity of each point from other classes. Summarizing SS of all data points enables determination of the level of separability between two classes. The range of SS is −1 to 1, with −1 representing the worst separability and 1 representing the best separability. Equations (8) and (9) illustrate how it is computed:

(8) $$a\left(i\right)=d_{E}\left(x_{i},\;c_{h}\right)\;b\left(i\right)=d_{E}\left(x_{i},c_{l}\right)\;x_{i}\in C_{h}\;C_{h}\neq C_{l}\;$$

(9) $$ss\left(i\right)=\frac{b\left(i\right)-a\left(i\right)}{max\left(a\left(i\right),b\left(i\right)\right)}\;SS=\frac{1}{K}\overset{K}{\underset{k=1}{\sum }}\frac{1}{N_{h}}\overset{N_{h}}{\underset{i=1}{\sum }}ss\left(i\right)$$

where a(i) is the distance between a feature vector ($x_{i}$) and a centroid of its own class, b(i) is the distance of $x_{i}$ to the centroid of the other class. *ss(i)* the single SS for a single-feature vector, and $N_{h}$ is the number of feature vectors in the $h^{th}$ class.

- Fisher’s linear discriminate analysis index (FLDI) [28]

FLDI can be applied to a multiclass problem, which is the ratio between the between-class and within-class scatter matrices, as shown in Equations (10)–(12). A larger FLDI implies greater separability.

(10) $$S_{b}=\sum_{i=1}^{c}{\left(\mu_{i}-\mu \right)}^{T}\left(\mu_{i}-\mu \right)\;$$

(11) $$S_{w}=\sum_{i=1}^{c}\sum_{j=1}^{N_{i}}{\left(x_{ij}-\mu_{i}\right)}^{T}\left(x_{ij}-\mu_{i}\right)$$

(12) $$FLDI=\frac{trace\left(S_{b}\right)}{trace\left(S_{w}\right)}\;$$

where $S_{b}$ is the between-class scatter matrix, $S_{w}$ is the within-class scatter matrix, c is the number of classes, *$N_{i}$* is the number of feature vectors in the $i^{th}$ class, *$\mu_{i}$* is the mean feature vector in the $i^{th}$ class, μ is the mean of all classes, and $x_{ij}$ is the $j^{th}$ feature vector in the $i^{th}$ class.

- Separability index (SI) [29]

The SI measures distances between the centroid of the ellipse of each class and the nearest class averaged across all motion classes, as formulated in Equation (13). The higher the SI, the more separability there is between classes.

(13) $$SI=\frac{1}{N}\overset{N}{\underset{i=1}{\sum }}min{\;}_{j=1,\;j\neq i}^{j=N}\frac{1}{2}\sqrt{{\left(\mu_{i}-\mu_{j}\right)}^{T}S_{i}^{-1}\left(\mu_{i}-\mu_{j}\right)}\;$$

where *N* is the number of motion classes; $\mu_{i}$ and $\mu_{j}$ are the centroids of $i^{th}$ class and *$j^{th}$* class, respectively; and $S_{i}^{-1}$ is the covariance of the $i^{th}$ class.

#### 2.4.2. Repeatability Index and Correlation Coefficients

To investigate the performance of a trained classifier on other calibration data, we calculated the repeatability index and correlation coefficients between training and testing calibration data. The change in feature space distribution can reflect the temporal and spatial variation in EMG signals [25]. Therefore, the selected correlation coefficients are primarily used to determine whether the distributions differ, indicating the consistency of the calibrations. We concatenated all channels for each motion to obtain each feature space’s kernel-smoothed probability density functions (PDFs). Subsequently, correlation coefficients were calculated based on PDFs. Equations (14)–(16)show these values were calculated. Because correlation coefficients are computed between two single-feature distributions, we averaged them over features and motions.

- Repeatability index (RI) [29]

The RI was previously explored in [29,30]. Both results showed that RI is an effective index to measure the consistency of EMG motion patterns in feature space generated in different trials. The RI is calculated as the distance between the centroid of the ellipse in one calibration and the class in another calibration, then averaged over all motion classes. It is formulated as in Equation (14).

(14) $$RI=\frac{1}{N}\overset{N}{\underset{i=1}{\sum }}\frac{1}{2}\sqrt{{\left(\mu_{Tri}-\mu_{Tsi}\right)}^{T}S_{Tri}^{-1}\left(\mu_{Tri}-\mu_{Tsi}\right)}$$

where *N* is the number of motion classes; $\mu_{Tri}$ and $\mu_{Tsi}$ are the centroid of $i^{th}$ training and testing class, respectively; and $S_{i}^{-1}$ is the covariance of the $i^{th}$ training class. A lower RI indicates more consistency between training and testing data.

- Two-Sample Kolmogorov–Smirnov Test statistics (K-S) [31]

K-S provides information on the similarity between two distributions as formulated in Equation (15). Data from training and testing tend to be well-correlated when the K-S is low.

(15) $$K-S=\frac{1}{N}\sum_{i=1}^{N}\frac{1}{M}\sum_{j=1}^{M}\underset{x_{i}}{\max}\left|F_{1}\left(x_{ij}\right)-F_{2}\left(x_{ij}\right)\right|\;$$

where $F_{1}\left(\cdot \right)$ and $F_{2}\left(\cdot \right)$ are the cumulative distribution functions of two feature distributions, M is the number of features in the feature space, and N is the number of motion classes.

- Spearman correlations (rho) [32]

Rho measures how two distributions are monotonically related. It is explained in Equation (16). In the rho value, −1 indicates that two feature distributions are totally different, whereas 1 represents the highest similarity between two feature distributions.

(16) $$rho=1-\frac{6\sum d^{2}}{n\left(n^{2}-1\right)}\;$$

where d is the rank difference between the two ranks of each probability density, and *n* is the number of probability densities.

### 2.5. Data Analysis

Linear discriminant analysis (LDA) was selected as the classifier. Classification can be divided into two parts. In the first part, called within-calibration classification (WCC), we used an eightfold cross-validation procedure to evaluate how the classifier performed when trained and tested within the same calibration. Another part estimated the between-calibration classification (BCC) performance using the leave-one-calibration-out cross-validation method. To determine whether there are statistically significant differences in classification performance between home and laboratory calibration data, we performed sign tests on both WCC and BCC errors. Furthermore, we applied linear regression between each separability index as an independent variable against WCC errors.

Similarly, linear regression was used between the repeatability index and each correlation coefficient as independent variables against BCC errors. The linearity between these indices and classification errors was represented by the *p*-value and R-squared value of each linear model to determine whether they are reasonable to describe calibration data viability. We used the sign test to determine statistical differences between home and laboratory settings for each evaluation metric.

## 3. Results

### 3.1. Raw sEMG Signals

**Figure 1:**
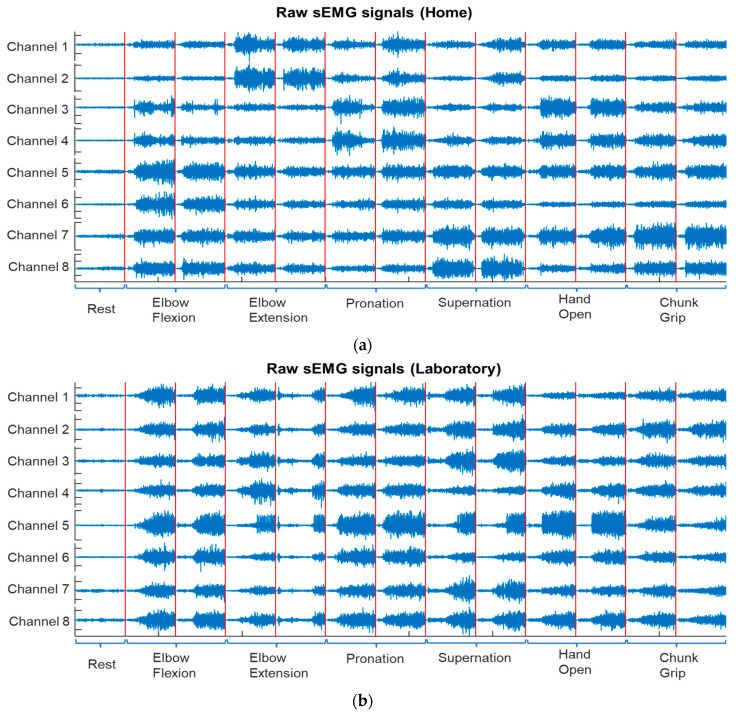
An example of TH02’s calibration data with all seven motions (**a**) at home and (**b**) in a laboratory setting. The red vertical lines separate different motions.

Figure 1 shows an example of the concatenated raw sEMG signals of seven motions of TH02 used to calibrate his prosthesis at home and in the laboratory.

### 3.2. Statistical Properties and Classification

**Figure 2:**
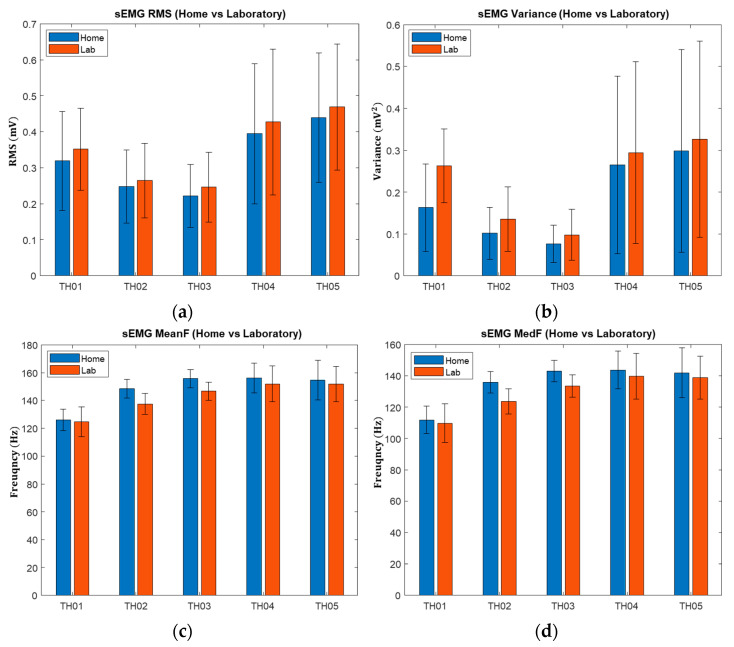
The average statistical properties of sEMG over the respective number of calibrations for each participant at home and in the lab: (**a**) root mean square values; (**b**) variances; (**c**) mean frequency; (**d**) median frequency.

The four statistical properties of sEMG from home and laboratory setting for each participant are shown in Figure 2. The sign test revealed a significant difference in the RMS and the variance of sEMG, which were both larger in the laboratory than at home (p<0.001). There was a greater mean and median frequency in the home than in the laboratory (*p* < 0.001). The sign test results for calibrations of all participants between home and laboratory are summarized in Table 2.

WCC and BCC errors are presented in Table 3. All BCC errors are larger than those of WCC, with the lowest error of 28.40 ± 4.91% for BCC and 5.61 ± 1.55% for WCC. The overall absolute value of the global mean WCC and BCC errors in the laboratory is higher than at home, although only BCC showed a significant difference (*p* < 0.05).

### 3.3. Metrics for Calibration Quality Quantification

**Figure 3:**
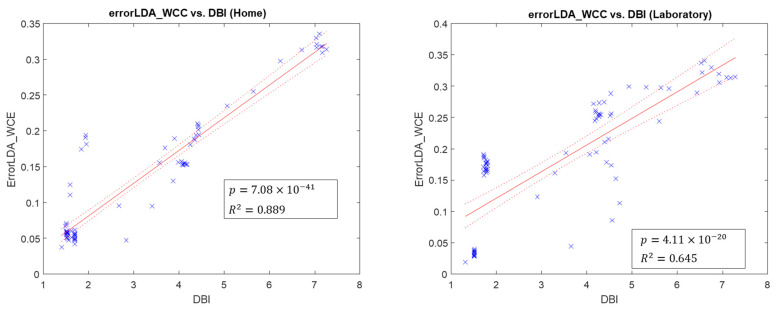
Scatter plots of linear regression fitted models (in red), where WCC error is related to DBI. Each blue x represents a sample result.

**Figure 4:**
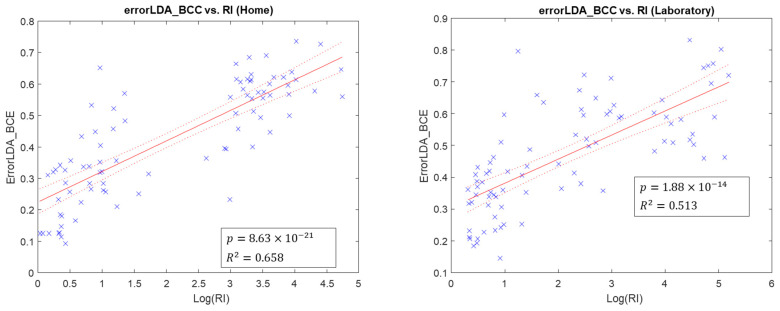
Scatter plots of linear regression fitted models (in red), where BCC error is related to RI. Each blue x represents a sample result.

For all metrics used to quantify the quality of signals, the line-fitting results across metrics and classification errors from all participants are summarized in Table 4. Figure 3 and Figure 4 show examples of how we fitted WCC with DBI and BCC with RI into linear regression models.

All separability indices have a high degree of linear relationship with WCC errors in home and lab contexts. WCC errors are lower with lower DBI and higher SS, FLDI, and SI. Additionally, RI has a linear relationship with BCC errors (higher RI with higher BCC errors) in home and lab calibration data. In contrast, K-S and rho have no and low linearity with BCC error in lab calibration data, respectively. Based on the averaged index values across all calibrations and the sign test on all metrics, only DBI and SI indicate that home calibrations have better separability than laboratory calibrations.

## 4. Discussion

The aim of this study was to compare the calibration of sEMG signals between home and laboratory settings through analysis of the statistical properties of sEMG signals and to quantify the calibration quality in both contexts. The overall results shows a better calibration quality at home than in the laboratory. In sEMG signals, RMS is related to the contraction forces, and variance represents sEMG signal power. Statistical analysis results show that there is a significant difference between home and laboratory settings, which as contraction levels vary between the two contexts. Because it is difficult for amputees to consistently produce contraction levels without proprioceptive and visual feedback [33], the force used to calibrate prostheses can vary each time. In the laboratory, amputees might have been more concentrated (i.e., high motivation or awareness) on performing motions, which resulted in high RMS and variance values. In addition, intensive concentration can lead to mental fatigue, which causes the recruitment of muscle fiber to be altered when generating the same force and motion pattern [34], which influences the consistency of the EMG signal. On the other hand, contraction levels could be estimated by Med F and Mean F, but the estimation is affected by the type of contraction, the subject, and the muscle length [35]. Med F and Mean F are the gold standards for assessing muscle fatigue using surface EMG signals because muscle fatigue results in a downward frequency shift [23]. Given the significant differences between home and laboratory setting in Med F and Mean F, muscle fatigue could potentially occur in the muscle when the participant calibrates their prosthesis in the laboratory.

**Figure 5:**
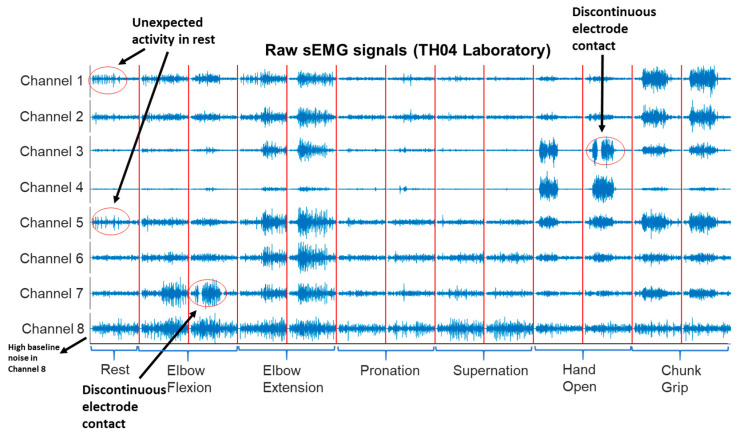
An example of laboratory sEMG signals with signal noise and user timing issues.

The WCC performance with the selected classifier and feature set obtained promising results with 6–8 weeks of home trial and lab calibration data. However, from the perspective of overall mean errors, the WCC errors in the lab are slightly higher than those in the home, despite no significant difference in the statistical test. In a study conducted by Waris et al. [8], LDA showed better performance and robustness than conventional classifiers on a fluctuated sEMG signal over seven days. Hence, the potential reason for the lack of difference in the WCC could be that the LDA and selected feature sets are robust to the divergence of sEMG between home and laboratory setting. During home-trial recording, signal noise and user timing issues could be the main reason for low-quality signals at home [19]. Signal noise issues include impedance change (when the skin’s temperature rises and sweat starts to form), intermittent electrode contacts with the skin (due to muscle volume variation when performing contraction, socket movement, etc.), and poor wire condition. User timing issues included unexpected activity during resting, insufficient contraction time, and missed contractions. Compared with home calibrations, calibrations in the lab also contained signal noise issues and timing issues, even under supervision. Figure 5 show a raw sEMG signal from the laboratory. In addition, we found that a large proportion of laboratory calibrations had issues of insufficient contraction time, which mixed resting signals with other motions. A short contraction time results in a low diversity between motion patterns and reduced classifiability. Furthermore, we used the resting-based threshold for WAMP to improve class separability [36]. The spontaneous activity during resting fluctuates the feature’s threshold and induces unknown motion into the signal.

On the other hand, BCC errors are much larger than WCC errors due to the stochastic characteristics of sEMG. Whereas we chose the calibration data as close in time as possible, the time interval between the two calibrations could be weeks, as the subjects calibrated the prosthesis at home for 6–8 weeks. The increasing time gaps between training and testing data deteriorated classification performance [5,8]. Except for TH04, all subjects had crossed home and laboratory trials or the interval was not more than one week. TH04’s lab trial was performed one month after the last calibration of the home trial. Because TH04 was not using the prosthesis for an extensive period, he could not produce consistent motion patterns across different calibrations in the lab. As a result, TH04 had the highest BCC error and, with a considerable difference in WCC error between home and lab settings.

In metrics for calibration quality quantification, DBI had the highest R-squared value, followed by SS and FLDI. With a reasonable degree of linearity, it can be concluded that these three indices can be used as quality indices to assist a user in determining whether additional calibrations for prostheses are needed. Because the repeatability index and correlation coefficient reveal the consistency between the two calibration data, they may compare calibration data with historical data with good motion patterns. Nathan et al. [37] developed a calibration quality feedback tool to increase the function of myoelectric prostheses. They used the separability index and repeatability index to evaluate calibration data with a rating system and advice for subsequent recalibration.

The results of this study are encouraging in terms of home use of myoelectric prostheses. However, the study is limited, as it only compares signals without considering contextual factors.

## 5. Conclusions

In this study, we adopted a dedicated methodological approach to assess the quality of data recorded at home during prosthesis use, data recorded in a laboratory setting, and how the two contexts affect performance. Results obtained in this study indicate that the within-calibration classification results of the sEMG of TMR amputees between home and laboratory settings did not significantly differ, but the quality of calibrations was different, with home data providing better separability. However, the between-calibration performance was better at home than in the laboratory despite no statistical difference in the repeatability metrics. These results show that although the motivation and engagement of patients might differ between home and laboratory settings, they have no significant influence on the within-calibration performance.

## Figures and Tables

**Table 1:** Participants enrolled in the dataset study.

Participant	Age	Time Since Amputation (Years)	Time Since TMR	Amputation Side	Etiology	Calibration Times	
						Home	Laboratory
TH01	35	4	3	Right	Trauma (military)	7	28
TH02	54	6	<1	Left	Trauma (military)	78	20
TH03	58	5	1	Left	Sarcoma	57	17
TH04	31	8	7	Left	Trauma (military)	22	25
TH05	27	2	1	Right	Trauma (crushing)	18	100

**Table 2:** The results of sign tests of calibrations for all participants in the laboratory and at home.

Statistical Property	Home	Laboratory	*p* Value
RMS	0.33 ± 0.11	0.35±0.11	$6.27\times 10^{-4}$
Variance	0.19 ± 0.12	0.22 ± 0.13	$4.08\times 10^{-5}$
Mean F	151.42 ± 10.81	145.16 ± 10.21	$8.54\times 10^{-9}$
Med F	138.70 ± 11.27	131.95 ± 11.15	$1.33\times 10^{-7}$

**Table 3:** Average classification error across calibrations.

Participant	WCC Error (%)		BCC Error (%)	
	Home	Lab	Home	Lab
TH01	5.61 ± 1.55	5.80 ± 3.60	28.40 ± 4.91	33.14 ± 12.47
TH02	6.72 ± 1.62	8.30 ± 3.14	21.10 ± 10.96	31.25 ± 10.94
TH03	7.77 ± 2.42	8.66 ± 3.33	40.85 ± 9.64	43.84 ± 13.79
TH04	6.73 ± 3.55	10.62 ± 4.37	54.49 ± 10.23	60.09 ± 10.36
TH05	4.84 ± 1.49	4.55 ± 2.78	58.22 ± 10.21	56.59 ± 13.19
Overall mean error (%)	6.33 ± 2.13	7.57 ± 3.44	40.61 ± 9.19	44.98 ± 12.15

**Table 4:** This table illustrates whether linearity exists (1) between separability indices and WCC errors and (2) between repeatability, correlation coefficient, and BCC errors. All R squares have *p* < 0.05, except for K-S in the laboratory. CC is the correlation coefficient. *p*-value indicates whether there are significant differences between the home and laboratory settings for each metric (bold-faced).

Metric		R-Squared Value		*p*-Value	Averaged Value across All Calibrations	
		Home	Lab		Home	Lab
**Separability indices**	DBI	0.89	0.65	0.011	**3.06 ± 1.87**	3.34 ± 1.87
	SS	0.81	0.84	0.063	0.31 ± 0.12	0.25 ± 0.14
	FLDI	0.86	0.72	0.156	−7.17 ± 2.86	−7.58 ± 2.68
	SI	0.54	0.85	0.012	**6.96 ± 5.64**	4.47 ± 2.99
**Repeatability index and CC ^1^**	RI	0.66	0.51	0.445	2.05 ± 1.48	2.16 ± 1.59
	K-S	0.29	0.00	0.156	0.19 ± 0.03	0.21 ± 0.04
	rho	0.46	0.12	0.913	0.89 ± 0.03	0.88 ± 0.04

^1^ N/A indicated that the results could not be determined because there are no significant differences.

## Data Availability

The data analyzed in this study are available from Levi Hargrove.
